# Association of State Medicaid Expansion With Hospital Community Benefit Spending

**DOI:** 10.1001/jamanetworkopen.2020.5529

**Published:** 2020-05-29

**Authors:** Genevieve P. Kanter, Bardia Nabet, Meredith Matone, David M. Rubin

**Affiliations:** 1 Division of General Internal Medicine, Department of Medicine, Perelman School of Medicine, University of Pennsylvania, Philadelphia; 2 Department of Medical Ethics and Health Policy, Perelman School of Medicine, University of Pennsylvania, Philadelphia; 3 Manatt, Phelps, and Phillips, LLP, Washington, DC; 4 PolicyLab, The Children’s Hospital of Philadelphia, Philadelphia, Pennsylvania; 5 Perelman School of Medicine, Department of Pediatrics, University of Pennsylvania, Philadelphia

## Abstract

**Importance:**

Medicaid expansion was widely expected to alleviate the financial stresses faced by hospitals by providing additional revenue in the form of Medicaid reimbursements from patients previously receiving uncompensated care. Among nonprofit hospitals, which receive tax-exempt status in part because of their provision of uncompensated care, Medicaid expansion could have released hospital funds toward other community benefit activities.

**Objective:**

To examine changes in nonprofit hospital spending on community benefit activities after Medicaid expansion.

**Design, Setting, and Participants:**

This cohort study used difference-in-differences analysis of 1666 US nonprofit hospitals that filed Internal Revenue Service Form 990 Schedule H detailing their community benefit expenditures between 2011 and 2017. The analysis was conducted from February to September 2019.

**Exposures:**

State Medicaid expansion between 2011 and 2017.

**Main Outcomes and Measures:**

Percentage of hospital operating expenditures attributable to charity care and subsidized care, bad debt (ie, unreimbursed spending for care of patients who did not apply for charity care), unreimbursed Medicaid spending, noncare direct community spending, and total community benefit spending.

**Results:**

Of 1478 hospitals in the sample in 2011, nearly half (653 [44.2%]) were small hospitals with fewer than 100 beds, and nearly 70% of hospitals (1023 [69.2%]) were in urban areas. Among the 1666 nonprofit hospitals, Medicaid expansion was associated with a decrease in spending on charity care and subsidized care (−0.68 [95% CI, −0.99 to −0.37] percentage points from a baseline mean [SD] of 3.6% [4.0%] of total hospital expenditures; *P* < .001) and in bad debt (−0.17 [95% CI, −0.32 to −0.01] percentage points). There was an increase in unreimbursed spending attributable to caring for Medicaid patients (0.85 [95% CI, 0.60 to 1.10] percentage points; *P* = .04), which canceled out uncompensated care savings from the expansion. Noncare direct community expenditures decreased overall (−0.24 [95% CI, −0.48 to 0.00] percentage points; *P* = .049). Direct community expenditures remained more stable in small hospitals (–0.07 [95% CI, –0.20 to 0.05] percentage points; *P* =.26) compared with large hospitals (–0.37 [95% CI, –0.86 to 0.12] percentage points; *P* = .14) and in nonurban hospitals (0.02 [95% CI, −0.09 to 0.14] percentage points; *P* = .70) compared with urban hospitals (–0.36 [95% CI, –0.73 to 0.01] percentage points; *P* = .06).

**Conclusions and Relevance:**

In this study, Medicaid expansion was associated with a decrease in nonprofit hospitals’ burden of providing uncompensated care, but this financial relief was not redirected toward spending on other community benefits.

## Key Points

**Question:**

Was Medicaid expansion associated with a change in nonprofit hospital spending on community benefits?

**Findings:**

In this cohort study of 1666 nonprofit hospitals using a difference-in-differences analysis, Medicaid expansion was associated with a 0.68-percentage point decline in spending on charity care and a 0.17-percentage point decline in bad debt. These declines in uncompensated care were offset by an increase of 0.85 percentage points in unreimbursed Medicaid-related spending, while noncare direct community spending decreased by 0.24 percentage points.

**Meaning:**

The results of this study suggest that although Medicaid expansion alleviated the financial stresses faced by hospitals in providing uncompensated care, these savings did not translate into additional direct community spending.

## Teaser

This cohort study examines changes in nonprofit hospital spending on community benefit activities after Medicaid expansion.

## Introduction

Medicaid expansion was widely expected to alleviate the financial stresses faced by nonprofit hospitals.^1,2^ A part of the Patient Protection and Affordable Care Act (ACA), Medicaid expansion provided federal financial support for states that expanded their Medicaid-eligible populations to include all adults with low income.^3^ Because nonprofit hospitals often provide, at a financial loss, free or heavily subsidized care to patients with low-income (so-called charity care), the expansion was expected to benefit hospitals by providing additional revenue in the form of Medicaid reimbursements from patients previously receiving uncompensated care.

Evaluations of the consequences of Medicaid expansion on hospitals have consistently shown that expansion decreased the financial deficits associated with uncompensated care.^4,5,6^ These findings suggest that Medicaid expansion could have important consequences for nonprofit hospitals in other areas of hospital activity. In particular, by driving down the need for delivering uncompensated care, expansion could affect hospital activities in their communities. The reason is that nonprofit hospitals must show that they engage in activities that provide benefits for the community and that improve community health to maintain their tax-exempt status; the delivery of uncompensated care has long been an important part of these community activities, collectively known as community benefits.^7^ By alleviating nonprofit hospitals’ uncompensated care burden, Medicaid expansion could release hospital resources for community benefit activities, thereby increasing hospital expenditures devoted to community benefits and improving public health.

Although federal law requires nonprofit hospitals to report their community benefit expenditures to the Internal Revenue Service (IRS), there is currently no established minimum community benefit requirement.^8^ Even without a mandated minimum, IRS reports show that pre-ACA community benefit spending in 2009 averaged 7.5% of hospital operating expenses, approximately 85% of which were in the form of charity care and other care services.^9^

Previous work has analyzed the consequences of the ACA and, using a pre-post design, reported little change in community benefit spending between 2010 and 2014.^10^ Another study using data through 2016 reported a decline in charity care and an increase in unreimbursed Medicaid spending associated with Medicaid expansion, but no change in overall community benefit spending.^11^

In this study, we unpack these broad patterns, decomposing community benefit spending into spending attributable to uncompensated care and spending attributable to noncare community activities. We examine changes in spending for charity care and subsidized care, spending for unreimbursed care of Medicaid patients, and bad debt (ie, unreimbursed care for patients who did not apply for charity care). We also examine heterogeneity in hospitals’ responses to the expansion. Using a difference-in-differences approach, we compare spending changes in hospitals in states that expanded Medicaid coverage between 2011 and 2017 with spending changes in hospitals in states that did not expand Medicaid.

## Methods

Per the Common Rule, this study was exempt from institutional review board approval because, as a study of hospital expenditures, it is not considered human participant research. This report conforms to the Strengthening the Reporting of Observational Studies in Epidemiology (STROBE) reporting guideline for cohort studies.

### Data

Since 2009, federal law has required nonprofit hospitals to annually file Form 990, which details their overall revenues and expenses, and Schedule H, which details the community benefit expenses and activities that provide justification for the hospital’s tax-exempt status.^12,13^ Schedule H includes information on expenses that federal law has recognized as constituting community benefits; these include, among other items, charity care (ie, free care provided to those whom the hospital has deemed eligible for financial assistance); unreimbursed Medicaid costs; subsidized care; community health improvement services; education for health professionals; and research. Schedule H also includes information on expenses for items that some hospitals and states consider community benefit but that the IRS does not recognize as such for federal tax exemption. These items include bad debt (ie, unreimbursed care for patients who did not apply for charity care) and community infrastructure building activities.^12^

We obtained Form 990 and Schedule H data for US hospitals with individual Schedule H filings from Candid (previously known as GuideStar) for 2010 to 2018. Candid digitizes data from publicly available tax filings of nonprofit organizations and makes these data available for purchase.^14^ Complete expenditure information was assembled for the 2011 to 2017 calendar years using filings from 2010 to 2018 (hospitals varied in their filing dates and used a range of fiscal years that sometimes crossed calendar years).

We linked hospital-specific expenditure data obtained from Form 990 and Schedule H to data on hospital characteristics obtained from annual American Hospital Association surveys. Data on social, economic, and demographic characteristics were obtained from the US Bureau of Census annual American Community Surveys. These zip code–level data were aggregated to the health service area–level by averaging across zip codes in each health service area and weighting by the population in each zip code. Hospitals were linked to the characteristics of the health service area that included the zip code of their physical location.

The final analytic sample consisted of 1666 acute care hospitals in 50 US states and the District of Columbia that filed at least 1 Schedule H form reporting on expenditures between 2011 and 2017. Because not all hospitals reported for all years, this sample was an unbalanced panel.

### Medicaid Expansion

Following precedent in medical and health services literature on Medicaid expansion, we used a binary variable to indicate Medicaid expansion and considered a state to have expanded Medicaid if it expanded its Medicaid-eligible population through executive or legislative action via the ACA mechanism or a Section 1115 waiver.^6^ The month and year of the implementation of Medicaid expansion for each state was obtained from legislative summaries produced by the Kaiser Family Foundation.^15^ States were considered to have expanded in a given year during the 2012 to 2017 period if expansion was implemented on or before June of that year. Following recommended practice,^16^ we excluded states that expanded Medicaid coverage before 2011 because these states may have been on different trend paths than states that participated in the main expansion. The list of excluded pre-2011 expansion states, 2012 to 2017 expansion states, and comparison states may be found in the eTable in the Supplement. As a robustness check, we also conducted an analysis excluding expansion and nonexpansion states known to have generous eligibility requirements (ie, Delaware, Massachusetts, New York, Vermont, and Wisconsin).

### Outcomes

We analyzed total community benefit expenditures as a percentage of total operating expenditures, as reported in Schedule H. Other outcomes included the individual components of community benefits, aggregated into the 3 following categories: (1) charity care expenditures and subsidized care services; (2) Medicaid shortfall (ie, unreimbursed expenditures associated with providing care to Medicaid patients); and (3) noncare community-directed expenditures, which included community health improvement services, health professions education, research, and cash and in-kind contributions. We also analyzed bad debt, an expenditure reported on Schedule H that is recognized by some states but not by federal law as constituting community benefits. All outcomes were reported as a percentage of total hospital operating expenditures.

### Statistical Analysis

We estimated the consequences of Medicaid expansion using a difference-in-differences approach applied to the case where treatment exposure (Medicaid expansion) occurred at different times for different units (hospitals).^16,17,18^ The model specification in the case of variable timing of treatment was as follows: Y_h,s,t_ = λD_h,s,t_ + X_h,s,t_β + α_s_ + γ_t_ + ε_h,s,t_, in which *Y* is the outcome of interest, *D_h,s,t_* is a treatment indicator for hospital *h* in state *s* in year *t* (which assumes the value of 1 when state Medicaid expansion is in effect for that hospital and 0 otherwise), *X* is a vector of covariates, *α_s_* is a vector of state fixed effects, *γ_t_* is a vector of year fixed effects, and *ε_h,s,t_* is the error term. The coefficient on the treatment indicator is the estimate of the association between expenditures and Medicaid expansion.

In the unadjusted difference-in-differences models, the vector of covariates *X* was omitted from the regression. In the adjusted models, the covariate vector was included and consisted of hospital characteristics (ie, number of beds, percentage of patients insured by Medicaid, percentage of patients insured by Medicare, urban location) and community characteristics (ie, percentage of population self-identifying as African American, percentage of population with less than high school education, percentage of population unemployed).

We also estimated event study models that captured the dynamic evolution of expenditures before and after Medicaid expansion. The specification for those models was as follows: Y_h,s,t_ = λ_1_D_h,s,T − 2_ + λ_2_D_h,s,T − 1_ + λ_3_D_h,s,T_ + λ_4_D_h,s,T + 1_ + λ_5_D_h,s,T + 2_ + λ_6_D_h,s,T + 3_ + X_h,s,t_β + α_s_ + γ_t_ + ε_h,s,t_, in which *D* was the treatment (expansion) indicator; *λ_1_*, the difference in outcomes between expansion and nonexpansion states 2 years before expansion year *T*; *λ_2_*, the difference between expansion and nonexpansion states 1 year before expansion; *λ_3_*, the difference between expansion and nonexpansion states during the initial Medicaid expansion year *T*; *λ_4_*, the difference between expansion and nonexpansion states during the first year after Medicaid expansion; and so on. The omitted year is 3 years before expansion.

This specification disaggregates the overall Medicaid expansion association during the first 4 treatment years to capture dynamic patterns. It also allows for a test of the parallel pre-expansion trends assumption. If expenditures in expansion and nonexpansion hospitals were on the same trajectory in the years before expansion, the coefficients in these pre-expansion years (ie, λ_1_ and λ_2_) should not be different from 0.

Models were estimated for the sample with all hospitals. Because hospitals could vary in their expenditure responses based on the sophistication of their administrative systems, we stratified by hospital size (<100 beds vs ≥100 beds), which served as a proxy for administrative capabilities. Because of differences in community needs between urban and rural areas, we also conducted analyses stratified by location. Significant associations were found for hospital size and urban location in formal tests of effect modification (results available upon request). Standard errors were clustered at the hospital level in all models.

Analyses was conducted from February to September 2019, using Stata version 16 (StataCorp). Statistical significance was set at *P* < .05, and all tests were 2-tailed.

## Results

The sample consisted of 1666 acute-care hospitals and a total of 9110 hospital-years (Table 1). Of 1478 hospitals in the sample in 2011, nearly half (653 [44.2%]) were small hospitals with fewer than 100 beds, and nearly 70% of hospitals (1023 [69.2%]) were in urban areas. The mean (SD) percentage of discharges attributable to Medicaid-insured patients was 17.3% (9.1%) in 2011 and 17.6% (9.4%) for the full study period.

**Table 1.** Descriptive Statistics of Hospital Sample

Characteristic	2011 (n = 1478 hospitals)	Full panel, 2011-2017 (n = 9110 hospital-years)^a^
Beds, No. (%)		
<100	653 (44.2)	4251 (46.7)
100-299	524 (35.4)	3072 (33.7)
≥300	301 (20.4)	1787 (19.6)
Discharges attributable to Medicare-insured patients, mean (SD), %	51.6 (12.1)	52.4 (12.3)
Discharges attributable to Medicaid-insured patients, mean (SD), %	17.3 (9.1)	17.6 (9.4)
Urban location**, **No. (%)	1023 (69.2)	6159 (67.6)
African American population, mean (SD), %	9.4 (13.1)	9.4 (13.1)
Population with <high school education, mean (SD), %	14.3 (5.7)	13.2 (5.5)
Population unemployed, mean (SD), %	8.3 (2.9)	8.0 (3.1)
Total operating expenditures, mean (SD), in millions of US $	194.7 (309.8)	224.0 (434.2)
Total community benefit expenditures, mean (SD), % of total operating expenditures^b^	7.8 (5.0)	8.1 (5.1)
Charity care and subsidized care expenditures, mean (SD), % of total operating expenditures	3.6 (4.0)	3.6 (3.9)
Medicaid shortfall, mean (SD), % of total operating expenditures	3.1 (3.0)	3.4 (3.4)
Community-directed expenditures, mean (SD), % of total operating expenditures	1.1 (2.0)	1.1 (2.8)
Unreimbursed care expenditures, mean (SD), % of total operating expenditures	0.7 (1.8)	0.8 (2.0)

^a^ Statistics for full panel are counts and averages over all hospital-years for all years (2011-2017). A total of 1666 hospitals were included.

^b^ Excludes bad debt, which is not federally recognized as part of community benefit expenditures.

Mean (SD) total operating expenditures were $224.0 ($434.2) million when averaged over the study period. Mean (SD) total community benefit expenditures were 8.1% (5.1%) of total operating expenditures, with 3.6% (3.9%) attributable to charity care and subsidized care expenditures and 1.1% (2.8%) attributable to noncare community-directed expenditures.

Between 2012 and 2017, 26 states expanded their Medicaid programs, most of which expanded in 2014 (eTable in the Supplement). Overall 1034 hospitals (62.0%) in the sample were located in expansion states. The remainder were in 19 states that had not expanded Medicaid by the end of 2017.

Table 2 reports the mean (SD) percentage of hospital expenditures attributable to total community benefit spending and to the 4 main categories of spending in 2011 in states that did and did not expand Medicare during the study period. In both groups, mean (SD) charity care and subsidized care spending accounted for 3% to 4% of hospital spending (expansion states 3.3% [3.3%]; nonexpansion states, 4.3% [4.9%]), approximately the same share as the mean (SD) Medicaid shortfall (expansion states, 3.4% [3.2%]; nonexpansion states 2.6% [2.5%]). Bad debt constituted less than 1% of hospital expenditures (mean [SD] in expansion states, 0.6% [1.9%]; nonexpansion states, 0.8% [1.7%]). Community-directed spending accounted for approximately 1% of hospital spending (mean [SD] in expansion sates, 1.3% [2.3%]; nonexpansion states, 0.9% [1.4%]).

**Table 2.** Association of Medicaid Expansion With Community Benefit Expenditures, All Hospitals, 2011-2017

Community benefit expenditures	Proportion of operating expenses in 2011, mean (SD), %		Change, percentage points (95% CI)	
	Hospitals in expansion states (n = 927)	Hospitals in nonexpansion states (n = 551)	Unadjusted model coefficient	Adjusted model coefficient
Total community benefit expenditures^a^	7.9 (5.2)	7.6 (4.6)	0.09 (–0.30 to 0.48)	0.01 (–0.38 to 0.40)
Charity care and subsidized care	3.3 (3.3)	4.3 (4.9)	–0.63 (–0.93 to –0.34)^b^	–0.68 (–0.99 to –0.37)^b^
Bad debt (other unreimbursed care)	0.6 (1.9)	0.8 (1.7)	–0.13 (–0.29 to 0.02)^c^	–0.17 (–0.32 to –0.01)^d^
Medicaid shortfall	3.4 (3.2)	2.6 (2.5)	0.90 (0.65 to 1.15)^b^	0.85 (0.60 to 1.10)^b^
Community-directed expenditures	1.3 (2.3)	0.9 (1.4)	–0.22 (–0.46 to 0.01)^c^	–0.24 (–0.48 to 0.00)^d^
Net gain or loss from changes in uncompensated care and Medicaid shortfall combined^e^	NA	NA	–0.14	0.00

Abbreviation: NA, not applicable

^a^ Excludes bad debt, which is not federally recognized as part of community benefit expenditures

^b^ *P* < .001.

^c^ *P* < .01.

^d^ *P* < .05.

^e^ Net gain or loss was calculated as absolute value of the change in charity care + subsidized care + bad debt − Medicaid shortfall. Analysis based on full unbalanced panel of 1666 hospitals and 9110 hospital-years. All estimates were computed using difference-in-differences methods with standard errors clustered at the hospital level. The unadjusted model includes state and year fixed effects. The adjusted model includes state and year fixed effects, the percentage of hospital discharges attributable to Medicaid, the percentage of hospital discharges attributable to Medicare, the percentage of African American residents in the surrounding community (ie, hospital service area), the percentage of individuals with less than a high school education in the surrounding community, the percentage of unemployed individuals in the surrounding community, an indicator of whether the hospital is in an urban area, and categorical indicators of the number of hospital beds.

Table 2 also reports the unadjusted difference-in-differences estimates of the association between Medicaid expansion and expenditures as well as adjusted estimates. The unadjusted and adjusted models give very similar estimates. In the adjusted model, Medicaid expansion was associated with 0.68 (95% CI, 0.37 to 0.99) percentage point decrease in charity care and subsidized care spending (*P* < .001) and a 0.17 (95% CI, 0.01 to 0.32; *P* = .04) percentage point decrease in bad debt. At the same time, expansion was associated with an increase in the Medicaid shortfall (0.85 [95% CI, 0.60 to 1.10] percentage points; *P* < .001). Thus, declines in uncompensated care (ie, the sum of charity care, subsidized care, and bad debt) were fully offset by an increase in unreimbursed Medicaid expenditures. This calculation of net gain (or loss) in unreimbursed care spending is shown in Table 2. Noncare community spending declined by 0.24 (95% CI, 0.00 to 0.48; *P* = .049) percentage points.

Dynamic associations can be seen in the Figure, which shows differences in expenditures between hospitals in expansion states and those in nonexpansion states in years before and after Medicare expansion. In the years before expansion, there was little difference between the 2 sets of hospitals in any expenditure outcome. This provides strong evidence in support of the assumption of parallel trends.

**Figure.**
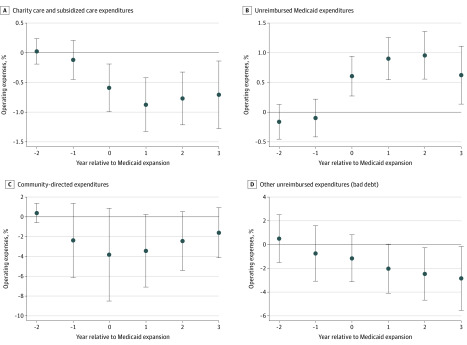
Association of Medicaid Expansion With Community Benefit Expenditures During Study Period Point estimates of dynamic associations and 95% CIs (whiskers) reported.

However, in the initial year of Medicaid expansion, charity care and subsidized care decreased significantly in hospitals in expansion states compared with those in nonexpansion states (−0.70 percentage points; 95% CI, −1.14 to −0.26; *P* = .002) (Figure, A). At the same time, unreimbursed Medicaid expenditure significantly increased (0.63 percentage points; 95% CI, 0.26 to 1.00; *P* = .001) (Figure, B). These unreimbursed expenditures continued increasing with each successive expansion year, tapering off slightly 3 years postexpansion.

In contrast, there is little statistically significant change in noncare community-directed expenditures (Figure, C), although the point estimates suggest that the expansion was associated with a large initial drop that subsequently abated. Declines in bad debt associated with Medicaid expansion followed a different pattern, increasing with each successive expansion year (Figure, D).

Table 3 shows that small hospitals (<100 beds) experienced a 0.87 (95% CI, 0.44 to 1.30) percentage point decline in charity care and subsidized care (*P* < .001), which was less than the 1.14 (95% CI, 0.71 to 1.56) percentage point increase in unreimbursed Medicaid expenditures (*P* < .001). Combined with bad debt, this resulted in a net loss of 0.35 percentage points. In contrast, large hospitals experienced roughly equal offsetting changes in charity care and subsidized care and Medicaid shortfalls. Overall, large hospitals experienced a net gain of 0.41 percentage points. Direct community expenditures remained more stable in small hospitals (–0.07 [95% CI, –0.20 to 0.05] percentage points; *P* = .26) than in large hospitals (–0.37 [95% CI, –0.86 to 0.12] percentage points; *P* = .14).

**Table 3.** Association of Medicaid Expansion With Community Benefit Expenditures, Small vs Large Hospitals, 2011-2017

Community benefit expenditures	Proportion of operating expenses in 2011, mean (SD), %		Change, percentage points (95% CI)	
	Hospitals in expansion states	Hospitals in nonexpansion states	Unadjusted model coefficient	Adjusted model coefficient
**Hospitals with <100 beds**				
No.	384	269	NA	NA
Total community benefit expenditures^a^	7.7 (6.0)	7.7 (4.9)	0.04 (–0.57 to 0.65)	0.07 (–0.54 to 0.69)
Charity care and subsidized care	3.5 (4.0)	4.3 (3.9)	–0.87 (–1.29 to –0.45)^b^	–0.87 (–1.30 to –0.44)^b^
Bad debt, ie, other unreimbursed care	0.7 (1.4)	0.9 (2.1)	0.07 (–0.16 to 0.30)	0.08 (–0.16 to 0.32)
Medicaid shortfall	3.5 (3.5)	2.8 (2.8)	1.13 (0.71 to 1.55)^b^	1.14 (0.71 to 1.56)^b^
Community-directed expenditures	0.6 (0.9)	0.6 (1.5)	–0.08 (–0.21 to 0.05)	–0.07 (–0.20 to 0.05)
Net gain or loss from changes in uncompensated care and Medicaid shortfall combined^c^	NA	NA	–0.33	–0.35
**Hospitals with ≥100 beds**				
No.	543	269	NA	NA
Total community benefit expenditures^a^	8.1 (4.5)	7.7 (4.9)	0.07 (–0.43 to 0.57)	–0.08 (–0.58 to 0.42)
Charity care and subsidized care	3.1 (2.7)	4.3 (3.9)	–0.45(–0.88 to –0.01)^d^	–0.55 (–1.00 to –0.10)^d^
Bad debt, ie, other unreimbursed care	0.6 (2.2)	0.9 (2.1)	–0.35 (–0.54 to –0.16)^b^	–0.40 (–0.60 to –0.21)^b^
Medicaid shortfall	3.2 (3.1)	2.8 (2.8)	0.61 (0.34 to 0.89)^b^	0.54 (0.26 to 0.82)^e^
Community-directed expenditures	1.7 (2.7)	0.6 (0.9)	–0.31 (0.77 to 0.15)	–0.37 (–0.86 to 0.12)
Net gain or loss from changes in uncompensated care and Medicaid shortfall combined^b^	NA	NA	0.19	0.41

Abbreviation: NA, not applicable.

^a^ Excludes bad debt, which is not federally recognized as part of community benefit expenditures.

^b^ *P* < .001.

^c^ Net gain or loss was calculated as absolute value of the change in charity care + subsidized care + bad debt – Medicaid shortfall. Analysis of small (<100 beds) hospitals based on unbalanced panel of 804 hospitals and 4251 hospital-years. Analysis of large (≥100 beds) hospitals based on unbalanced panel of 932 hospitals and 4859 hospital-years. All estimates were computed using difference-in-differences methods with standard errors clustered at the hospital level. The unadjusted model includes state and year fixed effects. The adjusted model includes state and year fixed effects, the percentage of hospital discharges attributable to Medicaid, the percentage of hospital discharges attributable to Medicare, the percentage of African American residents in the surrounding community (ie, hospital service area), the percentage of individuals with less than a high school education in the surrounding community, the percentage of unemployed individuals in the surrounding community, an indicator of whether the hospital is in an urban area, and categorical indicators of the number of hospital beds.

^d^ *P* < .05.

^e^ *P* < .01.

Nonurban hospitals experienced large declines in charity and subsidized care spending (−0.85 [95% CI, −1.37 to −0.34] percentage points; *P* = .001), no significant change in bad debt (0.05 [95% CI, −0.24 to 0.34] percentage points; *P* = .76), and large increases in Medicaid shortfall (1.07 [95% CI, 0.60 to 1.55] percentage points; *P* < .001), resulting in a small net loss (−0.17 percentage points) in unreimbursed care spending (Table 4). Urban hospitals experienced moderate declines in charity care and subsidized care spending (−0.59 [95% CI, −0.98 to −0.21] percentage points; *P* = .003), moderate declines in bad debt (−0.29 [95% CI, −0.47 to −0.11] percentage points; *P* = .01), and smaller increases in Medicaid shortfall (0.72 [95% CI, 0.44 to 1.00] percentage points; *P* < .001), resulting in a small net gain (0.16 percentage points) in unreimbursed care spending. At the same time, direct community spending stayed more constant in those nonurban hospitals experiencing net losses (0.02 [95% CI, −0.09 to 0.14] percentage points; *P* = .70) than urban hospitals (–0.36 [95% CI, –0.73 to 0.01] percentage points; *P* = .06).

**Table 4.** Association of Medicaid Expansion on Community Benefit Expenditures, Nonurban vs Urban Hospitals, 2011-2017

Community benefit expenditures	Proportion of operating expenses in 2011, mean (SD), %		Change, percentage points (95% CI)	
	Hospitals in expansion states	Hospitals in nonexpansion states	Unadjusted model coefficient	Adjusted model coefficient
**Nonurban hospitals**				
No.	266	189	NA	NA
Total community benefit expenditures^a^	7.1 (4.8)	7.3 (4.9)	0.29 (–0.38 to 0.97)	0.25 (–0.43 to 0.93)
Charity care and subsidized care	3.3 (3.5)	4.2 (3.9)	–0.80 (–1.31 to –0.29)^b^	–0.85 (–1.37 to –0.34)^b^
Bad debt (other unreimbursed care)	0.8 (1.4)	1.0 (2.0)	0.04 (–0.24 to 0.32)	0.05 (–0.24 to 0.34)
Medicaid shortfall	3.1 (3.2)	2.7 (2.9)	1.08 (0.59 to 1.56)^c^	1.07 (0.60 to 1.55)^c^
Community-directed expenditures	0.5 (0.9)	0.5 (1.0)	0.02 (–0.10 to 0.14)	0.02 (–0.09 to 0.14)
Net gain or loss from changes in uncompensated care and Medicaid shortfall combined^d^	NA	NA	–0.32	–0.17
**Urban hospitals**				
No.	661	362	NA	NA
Total community benefit expenditures^a^	8.3 (5.3)	7.7 (4.4)	–0.02 (–0.49 to 0.46)	–0.10 (–0.58 to 0.38)
Charity care and subsidized care	3.2 (3.2)	4.4 (5.3)	–0.54 (–0.91 to –0.16)^e^	–0.59 (–0.98 to –0.21)^e^
Bad debt (other unreimbursed care)	0.6 (2.1)	0.7 (1.6)	–0.25 (–0.42 to –0.07)^b^	–0.29 (–0.47 to –0.11)^b^
Medicaid shortfall	3.5 (3.3)	2.6 (2.3)	0.78 (0.50 to 1.06)^c^	0.72 (0.44 to 1.00)^c^
Community-directed expenditures	1.6 (2.6)	1.1 (1.5)	–0.33 (–0.69 to 0.02)	–0.36 (–0.73 to 0.01)
Net gain or loss from changes in uncompensated care and Medicaid shortfall combined^d^	NA	NA	0.01	0.16

Abbreviation: NA, not applicable.

^a^ Excludes bad debt, which is not federally recognized as part of community benefit expenditures.

^b^ *P* < .01.

^c^ *P* < .001.

^d^ Net gain or loss was calculated as absolute value of the change in charity care + subsidized care + bad debt – Medicaid shortfall. Analysis of nonurban hospitals based on unbalanced panel of 511 hospitals and 2951 hospital-years. Analysis of urban hospitals based on unbalanced panel of 1155 hospitals and 6159 hospital-years. All estimates were computed using difference-in-differences methods with standard errors clustered at the hospital level. The unadjusted model includes state and year fixed effects. The adjusted model includes state and year fixed effects, the percentage of hospital discharges attributable to Medicaid, the percentage of hospital discharges attributable to Medicare, the percentage of African American residents in the surrounding community (ie, hospital service area), the percentage of individuals with less than a high school education in the surrounding community, the percentage of unemployed individuals in the surrounding community, an indicator of whether the hospital is in an urban area, and categorical indicators of the number of hospital beds.

^e^ *P* < .05.

Our findings did not change when we excluded Delaware, Massachusetts, New York, and Vermont, states known to have generous eligibility requirements. The event study shows large decreases in charitable and subsidized care, offsetting increases in Medicaid shortfalls, and little change in direct community spending (eFigure in the Supplement).

## Discussion

Community benefit spending by nonprofit hospitals has come under scrutiny as lawmakers question whether these hospitals are contributing sufficiently to their communities to justify their tax-exempt status.^19^ With Medicaid expansion, we might have expected hospitals’ investments in their community to increase as nonprofit hospitals financially accrued gains from Medicaid reimbursements received for patients that they had previously treated at a financial loss. However, we found that growth of hospital revenue from Medicaid expansion did not translate into greater direct community spending.

We found that hospitals did experience declines in charity care with the implementation of Medicaid expansion. However, bad debt also diminished, suggesting that additional nonpaying patients who did not apply for charity care gained coverage through Medicaid expansion.

As has been reported elsewhere,^10,20^ declines in charity care were offset by increases in unreimbursed Medicaid expenditures. However, we also found that hospitals that experienced net gains (because declines in uncompensated care were greater than increases in unreimbursed Medicare expenses) did not necessarily increase their direct community spending, including urban hospitals and large hospitals, which experienced the greatest net gains.

One reason for the observed decline in community-directed spending may be that sophisticated hospitals capitalized on the influx of Medicaid patients—and the gap between Medicaid reimbursements and hospital charges—to enhance the appearance of total community benefit spending. Showing community benefit activities through changes in line-item reporting is certainly lower cost and less labor-intensive than carrying out direct community service programs.

A second, very different reason for the decline in community-directed spending might be increasing hospital costs; between 2007 and 2014, inpatient hospital prices increased by 42% and outpatient hospital prices by 25%.^21^ Thus, it is possible that even as hospitals received a net injection of dollars because care previously dispensed free of charge to uninsured patients was now being reimbursed by Medicaid, increasing costs of providing care may have outstripped any gains. However, a caveat is that the documented increases in hospital prices may not reflect true increases in the cost of providing care but rather increased market power (and profitability). The fact that some types of hospitals were able to maintain or increase their community-directed spending even as they experienced net losses in unreimbursed care suggests that increasing hospital costs may not be the full story.

### Limitations

There are several limitations to this analysis. First, as with all difference-in-differences analyses, it does not account for other changes that may have differentially occurred between expansion and nonexpansion states during this period. Second, our sample included only those hospitals that had filed individual 990 and Schedule H forms. Some hospitals that were part of health systems filed as separate entities and were included in our sample, but others were part of consolidated returns filed by health systems or universities and were not part of the sample. Although the hospitals in our sample represent approximately two-thirds of all acute-care nonprofit hospitals, the hospitals that did not file separately may engage in different patterns of community benefit spending. Finally, we used a binary indicator of Medicaid expansion, so we did not capture the myriad variations among states in the generosity and nature of the expansions. As a check on potential loss of information induced by use of this binary measure, we conducted an analysis that excluded states known to have generous eligibility requirements (ie, Delaware, Massachusetts, New York, Vermont, and Wisconsin). The event study figure for this robustness check is included in the eFigure in the Supplement and is very similar to results using the original sample.

## Conclusions

In this study, Medicaid expansion decreased nonprofit hospitals’ burden of uncompensated care but was not associated with noncare community benefit spending. That the financial gains from the expansion accrued by large and urban hospitals were not reflected in increases in community-directed spending should prompt closer scrutiny by lawmakers.

Although current law requires regular assessments of community health needs and reporting of community benefit spending, there is no established federal minimum standard for community benefit activity that is required for hospitals to maintain tax-exempt status. State standards for community benefit also vary widely across the country.^13^ Clearer, enforceable requirements for community-directed benefit spending alongside stronger financial incentives for population health through, eg, alternative payment models, might increase nonprofit hospitals’ direct investments in their communities.

## Notes

Supplement. **eTable. **States by Medicaid Expansion Status **eFigure.** Association of Medicaid Expansion With Community Benefit Expenditures Over Time Excluding States With Generous Pre-ACA Medicaid Benefits
