# Supplementary material for: Association of State Medicaid Expansion With Hospital Community Benefit Spending
Source: JAMA Netw Open. 2020 May 29;3(5):e205529. doi: 10.1001/jamanetworkopen.2020.5529 (PMC7260619; doi:10.1001/jamanetworkopen.2020.5529)
Supplement: Supplement. — eTable. States by Medicaid Expansion Status eFigure. Association of Medicaid Expansion With Community Benefit Expenditures Over Time Excluding States With Generous Pre-ACA Medicaid Benefits [file jamanetwopen-3-e205529-s001.pdf]

Supplementary Online Content

Kanter GP, Nabet B, Matone M, Rubin DM. Association of state Medicaid expansion with hospital community benefit spending. *JAMA Netw Open*. 2020;3(5): e205529. doi:10.1001/jamanetworkopen.2020.5529

eTable. States by Medicaid Expansion Status
eFigure. Association of Medicaid Expansion With Community Benefit Expenditures Over Time Excluding States With Generous Pre-ACA Medicaid Benefits

This supplementary material has been provided by the authors to give readers additional information about their work.

© 2020 Kanter GP et al. *JAMA Network Open.*

eTable. States by Medicaid Expansion Status

States that expanded prior to 2012 (excluded)	States that expanded between 2012 and 2017	States that had not expanded by end of 2017
Connecticut (4/2010)	Arizona (1/2014)	Alabama
District of Columbia (5/2010)	Arkansas (1/2014)	Florida
California (11/2010)	Colorado (1/2014)	Georgia
Washington (1/2011)	Delaware (1/2014)	Idaho (1/2020)
Minnesota (3/2011)	Hawaii (1/2014)	Kansas
New Jersey (4/2011)	Illinois (1/2014)	Maine (10/2019)
	Iowa (1/2014)	Mississippi
	Kentucky (1/2014)	Missouri
	Maryland (1/2014)	Nebraska
	Massachusetts (1/2014)	North Carolina
	Michigan (4/2014)	Oklahoma
	Nevada (1/2014)	South Carolina
	New Mexico (1/2014)	South Dakota
	New York (1/2014)	Tennessee
	North Dakota (1/2014)	Texas
	Ohio (1/2014)	Utah (1/2020)
	Oregon (1/2014)	Virginia (11/2018)
	Rhode Island (1/2014)	Wisconsin
	Vermont (1/2014)	Wyoming
	West Virginia (1/2014)	
	New Hampshire (8/2014)	
	Pennsylvania (1/2015)	
	Indiana (2/2015)	
	Alaska (9/2015)	
	Montana (1/2016)	
	Louisiana (7/2016)	

Source: Kaiser Family Foundation. Date in parentheses is the Medicaid expansion implementation date.

© 2020 Kanter GP et al. *JAMA Network Open.*

eFigure. Association of Medicaid Expansion With Community Benefit Expenditures Over Time Excluding States With Generous Pre-ACA Medicaid Benefits

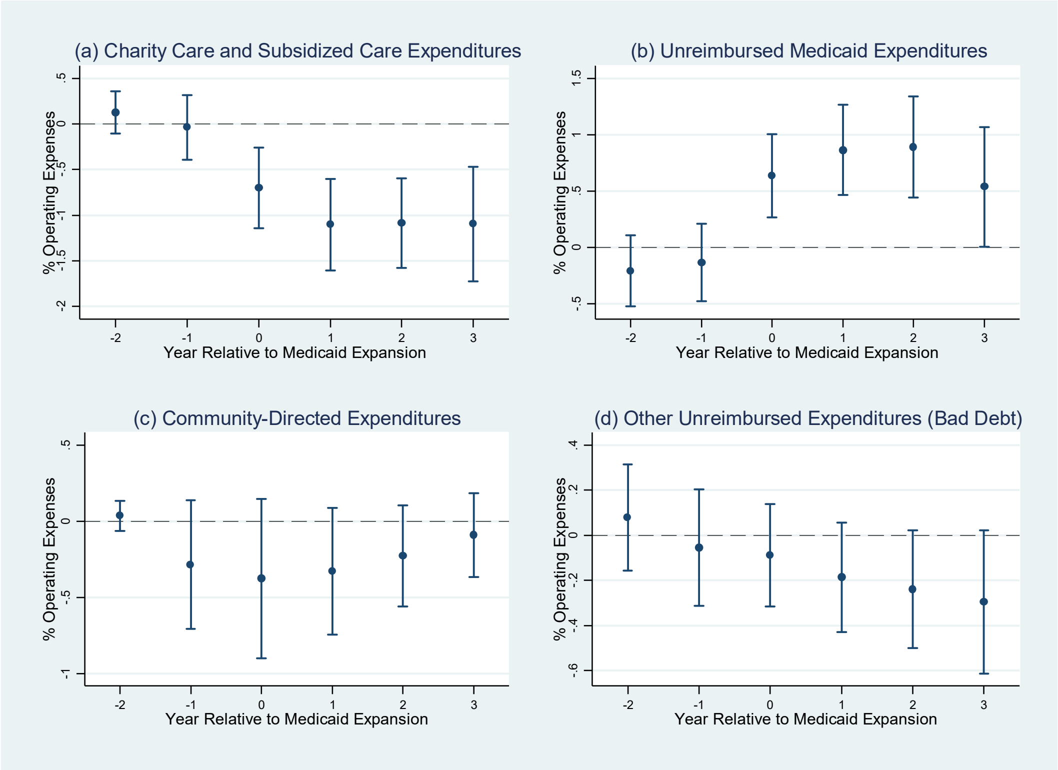

Point estimates of dynamic associations and 95% confidence intervals reported.

© 2020 Kanter GP et al. *JAMA Network Open.*
